# Supplementary material for: Complexome profiling of the Chlamydomonas psb28 mutant reveals TEF5 as an early PSII assembly factor
Source: Plant Cell. 2025 Mar 17;37(6):koaf055. doi: 10.1093/plcell/koaf055 (PMC12164590; doi:10.1093/plcell/koaf055)
Supplement: koaf055_Supplementary_Data [file koaf055_supplementary_data.zip › psb28_interactive.zip!/plots/intensity/qualityPlots/peptides/SCLB1_Cre17.g703700.t1.1.html]

### Cre17.g703700.t1.1

Trivial name: SCLB1  
SeqCoverage [median]: 42.80  
Mass: 44.48 kDa  
  
 

| Sequence | MissedCleavage | Mass [Da] | wtIntensity | muIntensity | Score |
| --- | --- | --- | --- | --- | --- |
| AVDQMADEKGEVVIK | 1 | 1630.82 | 2992.21 | 4241.98 | 93.42 |
| FGVNVPPGIPATTLDEVKK | 1 | 1981.09 | 3090.11 | 12596.78 | 75.91 |
| FTNGLQGGVHIVPK | 0 | 1465.80 | 0.00 | 620.22 | 40.48 |
| GKEILATSGMTIITADDLDDAAKK | 2 | 2476.27 | 0.00 | 2274.16 | 63.63 |
| LEGTNVAR | 0 | 858.46 | 6868.73 | 9892.14 | 119.29 |
| LGFDDNAAYR | 0 | 1140.52 | 3674.56 | 12463.95 | 134.05 |
| MHGGAPANFLDVGGSANEQQVVEAFK | 0 | 2672.27 | 584.66 | 0.00 | 90.10 |
| QMLGATLVTK | 0 | 1060.60 | 1340.44 | 0.00 | 65.31 |
| QVGVQVPLVVR | 0 | 1192.73 | 3678.30 | 12772.43 | 111.74 |
| SQILAGGR | 0 | 800.45 | 2010.65 | 3273.92 | 103.91 |
| VGITDAQAMQMVEGLR | 0 | 1717.85 | 649.22 | 2274.80 | 142.57 |
|  |  |  | 24888.9 | 60410.4 |  |
